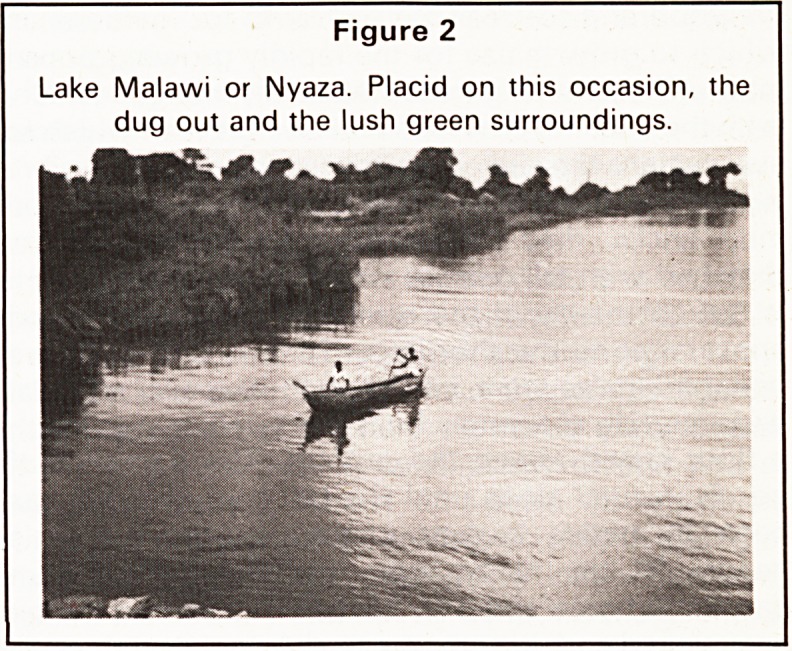# From Our Foreign Correspondents

**Published:** 1984-01

**Authors:** 


### From Our Foreign Correspondents

### An Orthopaedic Surgeon in Malawi

In the past 2 years I have spent 6 months in Malawi as a volunteer for Rotary International in their Lilongwe/M.A.P. Project, M.A.P. signifying 'Malawi against Polio'. Fortunately my wife Meriel has been able to accompany me on my last two tours and, on this last occasion, was a volunteer herself.

The preventive aspect of anterior poliomyelitis has been effectively dealt with by Save the Children Fund (Great Britain) with a nationwide mass immunisation programme and the Malawian Government Extended Immunisation Programme for the newborn. The supply of vaccine does run out and the cold chain can still break down (absence of paraffin for rural paraffin refrigerators is the commonest cause) but the paramount importance of prevention is fully appreciated and no effort is being spared on this front.

M.A.P. is a charitable organisation which began work in Blantyre in 1979 and has been operating increasingly successfully in the Southern region where about half of the six million population reside; it has had considerable financial support from Oxfam.

The new capital of Malawi is Lilongwe in the Central region which, with the smaller and more sparsely populated Northern region, comprises the other 50% of the population. The Rotary Club of Lilongwe, with praise worthy vision, applied to Rotary International in Illinois, U.S.A. for financial help to initiate a similar M.A.P. service in these two regions. A visit from Rotary International, including Professor Huckstep from Sydney, an expert in the attack on the situation left by anterior poliomyelitis in Africa from his work in Uganda, resulted in an agreement with the Malawian Government in which it would provide transport, three houses and some hospital beds and operating facilities, while Rotary International would contribute $220,000. This was the Lilongwe Rotary/M.A.P. Project launched to accomplish the aim of treating 2000 of the estimated 20,000 to 40,000 polio cripples within a span of 2 years.

The budgeting was detailed: 5000 calipers and clogs, 5000 crutches, 500 wheelchairs or tricycles, the visit of an orthopaedic surgeon for 4–5 weeks in each 3 months; Dr. Paul Binks, a very active Rotarian of 63 years about to retire from general practice in Ruislip as volunteer medical manager, with his wife Dr. Margaret Binks to help him; and Mr. Michael Ferry, a dynamic individual and, incidentally, a paraplegic confined to a wheelchair, of 16 years standing from an accident, as the Administrator of the project.

Mr. Ferry had been involved in a voluntary capacity in Blantyre with M.A.P. from its inception. He now moved to employment with the Project, bringing very valuable experience and assistance in liaison with the South. Paul and Margie Binks started work on 1st April 1981 and found the early weeks a trial, with the inevitable differences in interpretation of the agreement. Paul's wisdom, drive and pleasant personality overcame most difficulties, but when I arrived in June to start the clinical work we still had no beds in the ward in the old hospital in Lilongwe, but the workshop had started to manufacture calipers, clogs and crutches. We had one half-day operating session in the Kamuzu Central Hospital and one at the nearby Mission hospital in Likuni. Now hospitalisation is free in Malawi, but Mission hospitals have to make some charge to survive and this latter cost had therefore to be covered as an extra in the budget. We had two Ford Transit vans and with these travelled to newly opened clinics and even visited the Northern region over 100 miles of corrugated mud roads beyond the 130 miles of asphalt.

I had always refused to be circumscribed by the word 'polio' so that we included club feet up to adult life, cerebral spastic children, some tuberculous joints and various deformities. It was necessary to exclude the results of acute trauma or we would have been swamped. Excellent records were kept by Margie Binks and later by Meriel. We had most effective colleagues in the physiotherapists, Malawian and ex-patriate. Miss Gwen Gibbs, C.S.P. was a tower of strength in our work at Kamuzu Central Hospital, where we had our base and where she had for some years been the Government physiotherapist, supported by the Overseas Development Administration.

Three workshops have now been built up by M.A.P., the Rotary Workshop in Lilongwe referred to above, the Lions Club Workshop in Blantyre and the Prison Workshop in Zomba, this last only works well when there is an expatriate inmate, usually incarcerated after conviction of a crime related to uncontrolled avarice. These men usually succeed in running an excellent orthopaedic workshop in the prison.

Malawi now boasts 120 doctors and 100 of these are expatriates and most of these come from Britain. There is no Medical School in Malawi; of the 16 students sent to Manchester Medical School by the British Council very few have returned, most of them preferring to work in the N.H.S. for better salaries, better conditions, improved educational opportunities for their children and the chance in some cases of obtaining a higher qualification and to rank as a specialist on their return to Malawi. The Western world can be severely criticised for this loss of medical personnel from developing countries; it is too easy to stay away, get full registration and even British nationality.

Clinical Officers and Medical Assistants, two grades of medical manpower below Medical Officers, are trained in Malawi. An orthopaedic course lasting 3 months in 1981 had provided four Medical Assistants with the basis for becoming Orthopaedic Assistants after 2 years in service experience followed by a final course and an examination. These orthopaedic assistants are fundamental to the M.A.P. work and will undoubtedly play an even greater part in an orthopaedic service for Malawi; there is no orthopaedic surgeon for the six million people. A second course in 1982 brought the orthopaedic assistant numbers, trained and in training, to 10. Orthopaedic surgeons visiting Malawi in this Lilongwe Rotary/M.A.P. Project have come from the U.S.A. (4), India (1), South Korea (1) and I have done three tours. Those still in active practice can only get away for 4–5 weeks, while our last tour was $3\frac{1}{2}$ months to allow Drs. Paul and Margie Binks a well earned long leave, before accepting a third year. The Project was extended to a third year when more money was forthcoming from Rotary Clubs and from the Nuffield Foundation. Kamuzu Central Hospital was built by the Danish Government but, after constructing four theatres, the money was found to be running out and the builders could only build half the hospital which had been planned, so that we had only two theatres and two empty shells. The Ruislip and other Rotary Clubs have raised money completely to commission a third theatre (the orthopaedic theatre) and I did two full operating days each week in this theatre, the anaesthetists (clinical officers) and the nursing staff agreeing to service this theatre for the two days and they never failed me.

Sister Lua McDermott, C.S.P., of the Medical Mission of Mary from Eire, was invaluable over the many problems encountered at a clinic; our patients often had had no schooling; it cost money and fees increased in the higher classes, transport to school was often an insoluble problem and the clothes of these young people (up to early twenties) were more like rags as their poverty transcended even the average in the village scene. Sister Lua had to get them registered as disabled, which provided free education, if they could get to the school. Transport to hospital was usually by travel voucher and these had to be negotiated; communication with the patient at home was not possible unless someone among the family or friends could offer the P.O. Box number of their place of employment. Much had to be accomplished before the patient left the clinic.

It may well be asked what surgery was needed for a paralysed limb; older clinicians will know the answer as the orthopaedic clinical picture in Malawi today does resemble that in Britain in the late

**Figure 1**

The joy of attaining to the erect position.

twenties and early thirties – although much worse. In those days, contractures were a feature, the normal unopposed muscles producing over the years gross contracture of knees, hips and feet, anterior poliomyelitis much more commonly affecting the legs. I must admit that in Malawi much of the major surgery needed was for tuberculous spines, arthrogryposis and older club feet.

The surgery for M.A.P. in the Southern region is performed by the over-worked general surgeons and in the Central and Northern regions by visiting orthopaedic surgeons, volunteers for Rotary International, but these always spend 2 weeks of their time in the Southern region. The lesser surgical procedures are ably dealt with by Dr. Paul Binks and by the Orthopaedic Assistants in all regions. A lady surgeon, Dr. Schmidt, does excellent work in Zomba, the former capital, some 60 miles north of Blantyre. Thus the major orthopaedic surgery in M.A.P. is now being accomplished largely by visiting surgeons and out of this the orthopaedic service for Malawi must grow. The Government accepts the need for orthopaedic surgeons, one for Blantyre and the Southern region and one for Lilongwe to cover the Central and Northern regions. The Rotary International Project will have completed its third year at the end of March 1984 and "the Government must appoint an Orthopaedic Surgeon to continue the work.

The relationship of M.A.P., a voluntary body, to the Government Medical Services is already complex with polio patients being by no means a preserve of the former to the exclusion of the latter. The M.A.P. clinics are likely to become more and more the outreach orthopaedic clinics with M.A.P. and Government personnel continuing to work in harmony. The Orthopaedic Workshops, set up by M.A.P., are liable to be the main province into which the voluntary contributions to M.A.P. will continue to flow, but in so poor a country it is only too easy to withdraw a voluntary service only to find that the

Government, while fully recognising the need, is unable to take on the extra commitment from its diminutive budget. One must never forget that a recession hits the poorer countries even more severely than it does the richer ones.

I must now turn to our impression of Malawi as a country: it has beautiful scenery, an altitude ranging from 2000 to 5000 feet, apart from the lowlands of the lower Shire, with noble mountains, Mulanje in particular, rising to over 9000 feet with great appeal to mountain climbers as the highest peak in Central Africa (Kilimanjaro and Kenya being located in East Africa). The other outstanding beauty is Livingstone's lake, Lake Malawi, still called Lake Nyaza in Tanzania which also borders the lake and which sees no reason why one country should unilaterally change the name of a lake common to three countries! It is part of the Rift Valley, 350 miles long and 40–50 miles across. The numerous cataracts in the Shire river, which alone drains the lake and which were such a trial to David Livingstone in his frustrated efforts to get a steamer on to Lake Malawi in the early 1860s, have excluded contact between the fish of the lake and those elsewhere in the world. The result is a great number of beautiful fish, exclusive to these waters and these constitute an important export from a poor country.

The cataracts also provide the fall needed for the generation of electricity and it is from here that electricity is supplied throughout the country although it is only slowly being supplied to the distant Northern region. The Chamba is a delightful fish for the table but, as elsewhere, fishing with too fine a net is reducing numbers. The fishing boats are dugouts and one can see them being made on the lake shore. The lake can be beautifully placid or a raging sea, as when it resulted in the shipwreck and drowning of the first Bishop, Bishop Chauncy Maples, as he crossed to the island of Likoma where his Cathedral still languishes, so far from its diocese. Originally the missionaries worked mostly on the Eastern side of the lake, now Mozambique and Tanzania, and they built their Cathedral on the nearby island, not unreasonably, as transport was largely by water. The island and the Cathedral remain Malawian but lie so close to Mozambique and so far from mainland Malawi as to create a grotesque situation with poor prospects of any satisfactory resolution. The lake is beautiful throughout and the one lake steamer still carries the original name 'Ilala' given to the first Mission steamship on the lake, launched in 1876, 3 years after Livingstone's death in the district of that name.

The finest scenery is at the northern end where the towering Livingstone mountains rise vertically from the water's edge on the Tanzanian side. The slave routes to the Zanzibar slave market were either north of the lake or to the south, but a very important route was by dhow across the lake from N'Khota Kota to

**Figure 2**

Lake Malawi or Nyaza. Placid on this occasion, the dug out and the lush green surroundings.

Losefe, the former still has a lingering reputation in some Arabic blood and tradition in this languid township.

The lake continues to baffle the scientists as it can rise 10 feet or more over a decade, only to return to its former level in the subsequent 10 years. The hippopotami are still readily seen in the inlets leading to the lake, hyena are seen around Lilongwe and monkeys abound. The bird life is fascinating, particularly when you get away from the towns.

The altitude protects Malawi from the more severe droughts, as that which has overtaken so many African countries this year. Maize is the staple crop, grown by all but a few city dwellers in their 'gardens' as they refer to their plots of land, and all cultivated by hand with an enlarged Dutch hoe, with a blade about 12 inches wide and 6 inches deep. Only the large estates use the tractor and plough, some of these estates are owned by expatriate's concerns, but mostly by Malawian wealthy families or businesses, such as Press Holdings, which intricately involves the President personally and the State.

Tobacco is grown on large estates, but also on smallholdings, but tea, the other main export, is grown only on large estates. Cassava, a starchy root crop, is grown widely but is not satisfactory as a basic diet. Millet and some wet rice, grown round the lake, provide a valuable substitute or addition. The jacaranda, flame of the forest, acacia and African tulip tree have all been seen by us giving glorious colour to the trees and, nearer the ground, bougainvillea, the pointsetia, scarlet, pink and yellow and the aloes add to the beautiful scene in due season.

There is no mineral wealth in the country and wood and charcoal are the only indigenous alternatives to imported fossil fuels. The Australian blue gum is widespread and is grown in 'Fuel Forests' in urban areas, coppiced regularly and sold to the town people for firewood. In the more rural areas there exists a steady depletion of the trees to supply firewood and to clear more ground for 'gardens' in which to grow maize for the rapidly growing population. The surviving trees are usually mangos which, with the banana, provide so much fruit with which to supplement the diet. The houses in the country are wattle and daub or constructed of large sun-baked mud bricks, they are usually rectangular and are thatched with tall grasses which abound; the thatch is not cut neatly at the eaves, giving a dishevelled appearance to the dwellings. Each house will have its round maize store, constructed in similar material and standing separately from the dwellings and with a floor raised above the ground. The transport of goods in rural areas until the last two decades has been by native baskets carried on the women's heads. All the maize was thus transported from garden to home and only in recent years has the ox been yoked and, in some areas, the donkey trained to pull a cart. Consequently the carts all have old car wheels and are of similar vintage, clearly a relatively recent acquisition.

Travelling from Lilongwe, the new capital city, in the Central region to Mzuzu the principal town in the Northern region, one crosses the Vypia mountains and pass through hundreds of square miles of conifer forest. I was much impressed with this very successful afforestation, excellent in care of the trees, fire breaks and the progressive planting for cropping; but the trees are already too old for pulping and a late assessment of this development by Canadian experts has shown that it is quite uneconomic to pulp the timber and transport it to the nearest market and that the effluent from a pulping factory would ruin the lake. Thus we see conifers from horizon to horizon for many hours, driving laboriously over the mud roads, and the future of these trees remains in doubt – timber and fuel will no doubt be the answer, but the cost of transport again bedevils a country so long and so slender.

The Malawian people are very friendly and cheerful. When they have had good educational opportunities they are as able as most of us but, where the educated are not numerous and have known each other in their school days, one often finds a lack of leadership and organising ability – this is a criticism often heard in Malawi.

As in so many ex-colonial (protectorate in this case) countries in which I have worked, there is no resentment at this item in their history, some have even lamented the loss of the stability enjoyed in those days. The Malawians love colour in their dress and we enjoyed the bright colours, but poverty in the villages was great and patches often covered a greater area than the original garment.

Dancing is a cultural expression much enjoyed and widely practised, mostly by the women and featuring prominently in festivities.

The old capital Zomba has an old Residence from the days of the Governor of the Protectorate, but Blantyre has Sanjika Palace, built by the President and, in the new capital city, Lilongwe, there rises a further palace which has been 12 years to date in the building. These are features of the patriarchal regime of His Excellency Gwazi (the Wonderful) the Life President, Doctor H. Kamuzu Banda. I was invited to the State Banquet at the Sanjika Palace in Blantyre on the occasion of Independence Day celebrations (the 17th anniversary) in 1981. Lesser banquets took place at all the large hotels in the country and at 9 p.m. we were *all* listening to H.E.'s speech. At Sanjika Palace were seated 220 guests, all but 10 of us in dinner jackets or uniform; we had four wines, four courses, and soft music from a subdued military band played throughout until the speeches began. The glasses sparkled and the dishes were of quality and specially designed for the State Palace. The meal was served with military precision. Opposite me was the Chinese Ambassador and, in conversation, I had to be gently corrected – he was of course from Taiwan.

Was such an extravagant performance appropriate in so poor a country? Perhaps as an annual event to underline the dignity of Independence? I could not quite really subscribe to this conclusion although I fully appreciate the very important part played by pageantry in keeping a country contented, even at times enthusiastic with the regime.

Kamuzu Academy is the public school of 360 students selected, two from each district by examination results, which opened in 1981. It is clearly more public than those with this title in Britain. The buildings are as pleasing and well proportioned as any British public school and show H.E.'s excellent taste, but the school cost £10 million and has an entirely expatriate staff. The pupils in their boaters and uniforms are indeed a surprising encounter in Central Africa; this is an elitist conception of the President, hence his pronouncement that no Malawian teacher was fit to teach at the Academy, which remark sparked off a good deal of adverse criticism.

The pupils apply themselves assiduously to their work, but a rounded boarding school life is provided, with sport, dramatics, music and other ex-curricular activities—but where will the school leavers go? They will be too advanced for the modest Chancellor College University at Zomba and further education abroad will be very costly for such large numbers and can prove very wasteful when graduates fail to return.

There is only one President in Malawi, all other leaders of groups, societies or industries can aspire to no greater title than Chairman. The four words instilled into every Malawian are Unity, Loyalty, Obedience and Discipline, H.E.'s four principles for building a nation. The President approves all nominations for parliamentary election, these then have a course to improve their English, are shown to the electors but not allowed to make speeches 'lest they should be accused of canvassing for votes'. Finally, if elected as members of Parliament, they do not hold much sway. The Cabinet is chosen by the President and frequently changed by him. In spite of the firm hold on the nation imposed by the President, politicians have at times misused their power and they have been removed and punished and it was generally felt that justice was being done. The country was severely shaken this summer when four popular senior politicians, the General Secretary of the one Party, the Minister of Health, the Governor of the Central region and one other, were reported on the local radio one morning as being wanted by the police – 2 days later they were reported as all having died in a road accident. The funerals were private and incredulity was widespread – a struggle for the succession was clearly in progress.

In the past 10 years, passing as I do from the larger World developing countries back to Britain, I am sad to find so few interested in making a contribution to this great need. In the developing world I do meet doctors, physiotherapists and nurses who have opted to spend their lives there. Besides those in the mission field, there are many who are supported by the Overseas Development Administration of the Foreign and Commonwealth Ministry. There must be a teaching or training element in the work to obtain this support, which amounts to 'topping up' the local salary and assistance with education, the children among other things getting two return airfares a year when secondary education is being given in Britain. It is a challenging life and brings out the best in anyone. The spectrum of diseases is quite different but a good clinician will soon adapt: there is little hope for the doctor who relies too heavily on all those sophisticated aids to diagnosis, these will not be available to assist or even to replace his clinical judgement.

I meet some excellent people, medical, paramedical and others in these countries: there are 'Voluntary Service Overseas' personnel who work for minimal rewards but great experience; they do wonderful work, are as a rule young people and short term – a 2-year contract; there are many physiotherapists and some doctors in this group.

Then there are families with young children on 5 year contracts with the Government: it may not in the future be at all easy to enter General Practice in Britain from this background. Finally there are those who elect to do their life's work in these countries, meeting exacting demands but I have very rarely met anyone who did not enjoy the life and the challenge.

Many young doctors are now coming out from other European countries, where over production of doctors seems to have occurred even earlier than in Britain. Some go out as General Medical Officers, learn the language and are running a District Hospital within 6 months. They then return to their own country for postgraduate training to return in due course; the lure of Africa has held them and this is their vocation.

There is also the older doctor who has the advantage of having seen a somewhat similar spectrum of disease in his youth and, when he retires in Britain, he fits more readily into the picture. It can give great satisfaction and even lengthen his life!

The least contribution, in my opinion, is to attempt medical or surgical training in the Western World; it proves to be a wholly inappropriate training.

Malawi is a beautiful country, very poor as regards its ability to earn foreign currency and in the standard of living, a rural country with plenty of fruit and a reasonably secure harvest of cereals on which to survive. It needs our help and we do best in giving a market to its products and giving instruction and help *in* the country and not by tempting the talented Malawian to go abroad except for limited periods towards the end of his training – he will then return and build up the Malawi of the future.

ARTHUR L. EYRE-BROOK

o

**Letter from America by Alistair Look,** M.D.

The focal point in the clinical activity of an American University Teaching Hospital is the Staff Conference. The current approach to investigation and treatment can best be illustrated by a factual account of a lung cancer staging conference. Staging, randomisation, experimental models, statistical records and computer programming are the sacred cows of current surgical philosophy.

Wednesday morning and the Lung Cancer Staging Conference. First case. Male, 51, '2 pack a day for 40 years' (probably a gross understatement), tumour at right hilum, widening of the superior mediastinum and a nodule in left upper lobe. Computer tomography scan suggests involvement of the interbronchial lymph nodes and enlargement of the left adrenal. Bone marrow negative. Mediastinoscopy reveals adenocarcinoma in the right tracheobronchial lymph nodes. Primary or secondary? Barium follow through and enema to exclude bowel primary. Intravenous pyelogram and cholecystogram for good measure. Pulmonary function tests and differential perfusion study. Gallium scan reveals 'hot foci' at right hilum, left cerebral hemisphere second and tenth vertebral bodies. Ultrasound of pancreas inconclusive. Bronchoscopy? Nobody thought of doing it. Patient's symptoms? Nobody thought of asking him. Conclusion: Stage 3, T3, N3, M3. Plan: laparotomy for biopsy of left adrenal, needle biopsy of nodule in left upper lobe, jejunostomy to raise serum albumen. Problem: entry to the randomised protocol for DXT to brain plus chemotherapy; or DXT to right hilum, and chemotherapy if no response to 3000 rads. $36,000 dollars, so far. 'Interesting case'.

At this point the routine coffee and doughnuts arrive and an ugly rush ensues. The heaviest attenders get there first by dint of weight and numbers. Order is finally restored and the second case is presented.

Left upper lobectomy for a coin lesion, found to be a granuloma. Unfortunately the patient does not qualify for randomisation and is therefore of no interest. The fact that the patient has a post-thoracotomy empyema and an infected wound, and is still on a respirator in the ICU also arouses no comment.

Next case please. Right upper lobectomy and sleeve resection of the bronchus for a squamous cell hilar lesion 2 years previously. Returns now with haemoptysis, right hilar enlargement, pain in the back and dizzy spells. Bronchoscopy, performed with reluctance, reveals a squamous carcinoma in the right main bronchus. As it is 2 years since the first intervention, this is classified as a second primary rather than a local recurrence. Gallium scan of brain and spine not done. Plan: randomisation for DXT to right hilum, or chemotherapy, or both. As virtually all patients return to 'Marlboro country' within days of a lung resection for cancer the second primary theory may not be so fanciful.

The next patient has been eliminated from the conference as he has just died following an open lung biopsy, much to the amusement of the medical oncologists.

Next case please. The Senior Resident presenting the case has fallen asleep. He has been up all night attempting to salvage 2 cases of gunshot wounds to the heart. The second case was stone cold on arrival in the Emergency Room. But a median sternotomy and a video record of the heart, well ventilated by two or three .38 calibre bullets, is demanded by the Emergency Room Physicians, an independent corps unrelated to the Department of Surgery, for teaching purposes.

The staging conference continues. The doughnuts have disappeared, along with half the audience. A further 15 cases are reviewed. Of the 18 patients 'staged' during the conference, one may survive longer than 9 months, if he recovers from the empyema. The hopeless state of the majority of cases reviewed makes a mockery of early diagnosis, primary care or preventive medicine. Ancillary treatment is justified, in spite of the expense, by the prolongation of survival by an average of 2 months, irrespective of the fact that much of the survival time will be spent in hospital under treatment for the more serious complications of chemotherapy and radiotherapy. The futility of this philosophy has not yet been generally accepted. Failure to treat will probably result in a financially crippling malpractice suit.

I have been criticised for occasionally walking on the campus at night and for not carrying a handgun. Most of the nursing staff pack a .357 Magnum, or a tear gas spray, in their handbags. Supposing I shoot a 'mugger' in self-defence, what do I do? Beat it quickly. The police are overwhelmed by crimes of violence and will rarely make any attempt to trace the person responsible, especially if the attacker is a known criminal with a record. The greatest deterrent to the carrying of any 'hardware' for self-defence, legal or otherwise, is the risk of shooting the wrong person, a decent law-abiding citizen who might be trying to offer help or guidance. Unfortunately the mood of the muggers has changed from 'rob or kill' to 'rob and Kill'. It is doubtful if the handgun problem will be resolved within the foreseeable future. The constitutional amendment 'entitling the citizen to bear arms' is conveniently misquoted by omission of the rider 'in the defence of his country' rather than the protection of his life or property, or her virtue.

Elections for State Governors, Mayors or other civic officials are truly democratic. The motto is 'vote early and vote often'. Every change of domicile entitles a citizen to a new registration as a voter. A recent mayoral election produced a turnout of 80%, a national record. Not so satisfactory was the revelation that many of the 'voters' had been dead for several years. When the turnout approaches 120% of the register, the system may be 'looked at'.

America is still a great country. A cogent reason was recently emphasised by a survey of the popularity of the 'work ethic' or dedication to one's work and its accomplishment to the best of one's ability. Fifty-two per cent of Americans conformed to the work ethic as countrasted to 17% of British citizens. The economic and social consequences of this fundamental difference in philosophy are mind-boggling.

Further differences in surgical philosophy between the United States and Britain are partly explained by the American legal system. If the surgeon operates, he is sued for malpractice. If he refrains he is sued for negligence. Overmanning is becoming a serious problem, as in the rest of the world, and too many surgeons are chasing too little pathology is spite of heroic efforts to discover additional pathology. In one centre a routine policy of submitting every case to a second surgical opinion resulted in an immediate drop of 20% in the number of surgical procedures performed, with no detriment on long-term follow-up to those patients reprieved. Difficult to understand is the apparent willingness of the average American patient to submit to multiple surgical procedures for the same problem. Re-do surgery, often of extreme technical complexity, is now the major exercise of the sophisticated surgical departments of University teaching centres. Continual teaching programmes, meetings, conference and refresher courses have had little impact on this problem and how to teach the majority of the profession how to do it right the first time and eliminate the necessity for revision surgery is now the most pressing challenge to the teaching centres.

## Figures and Tables

**Figure 1 f1:**
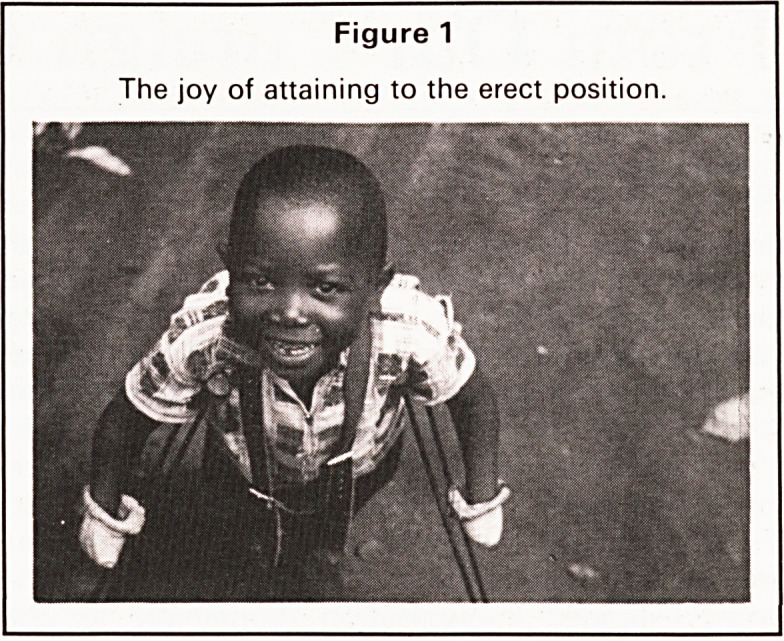


**Figure 2 f2:**